# Complex Quantum Network Manifolds in Dimension *d* > 2 are Scale-Free

**DOI:** 10.1038/srep13979

**Published:** 2015-09-10

**Authors:** Ginestra Bianconi, Christoph Rahmede

**Affiliations:** 1 School of Mathematical Sciences, Queen Mary University of London, London E1 4NS, United Kingdom; 2 Institute for Theoretical Physics, Karlsruhe Institute of Technology, 76128 Karlsruhe, Germany

## Abstract

In quantum gravity, several approaches have been proposed until now for the quantum description of discrete geometries. These theoretical frameworks include loop quantum gravity, causal dynamical triangulations, causal sets, quantum graphity, and energetic spin networks. Most of these approaches describe discrete spaces as homogeneous network manifolds. Here we define Complex Quantum Network Manifolds (CQNM) describing the evolution of quantum network states, and constructed from growing simplicial complexes of dimension 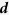. We show that in ***d ***= 2 CQNM are homogeneous networks while for ***d ***> 2 they are scale-free i.e. they are characterized by large inhomogeneities of degrees like most complex networks. From the self-organized evolution of CQNM quantum statistics emerge spontaneously. Here we define the generalized degrees associated with the 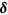-faces of the 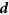-dimensional CQNMs, and we show that the statistics of these generalized degrees can either follow Fermi-Dirac, Boltzmann or Bose-Einstein distributions depending on the dimension of the 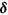-faces.

Several theoretical approaches have been proposed in quantum gravity for the description and characterization of quantum discrete spaces including loop quantum gravity^1^,^2^,^3^, causal dynamical triangulations^4^,^5^, causal sets^6^,^7^, quantum graphity^8^,^9^,^10^, energetic spin networks^11^,^12^, and diffusion processes on such quantum geometries^13^. In most of these approaches, the discrete spaces are network manifold with homogeneous degree distribution and do not have common features with complex networks describing complex systems such as the brain or the biological networks in the cell. Nevertheless it has been discussed^14^ that a consistent theory of quantum cosmology could also be a theory of self-organization^15^,^16^, sharing some of its dynamical properties with complex systems and biological evolution.

In the last decades, the field of network theory^17^,^18^,^19^,^20^,^21^ has made significant advances in the understanding of the underlying network topology of complex systems as diverse as the biological networks in the cell, the brain networks, or the Internet. Therefore an increasing interest is addressed to the study of quantum gravity from the information theory and complex network perspective^22^,^23^.

In network theory it has been found that scale-free networks^24^ characterizing highly inhomogeneous network structures are ubiquitous and characterize biological, technological and social systems^17^,^18^,^19^,^20^. Scale-free networks have finite average degree but infinite fluctuation of the degree distribution and in these structures nodes (also called “hubs”) with a number of connections much bigger than the average degree emerge. Scale-free networks are known to be robust to random perturbation and there is a significant interplay between structure and dynamics, since critical phenomena such as in the Ising model, synchronization or epidemic spreading change their phase diagram when defined on them^25^,^26^.

Interestingly, it has been shown that such networks, when they are evolving by a dynamics inspired by biological evolution, can be described by the Bose-Einstein statistics, and they might undergo a Bose-Einstein condensation in which a node is linked to a finite fraction of all the nodes of the network^27^. Similarly evolving Cayley trees have been shown to follow a Fermi-Dirac distribution^28^,^29^.

Recently, in the field of complex networks increasing attention is devoted to the characterization of the geometry of complex networks^30^,^31^,^32^,^33^,^34^,^35^,^36^,^37^,^38^,^39^. In this context, special attention has been addressed to simplicial complexes^40^,^41^,^42^,^43^,^44^, i.e. structures formed by gluing together simplices such as triangles, tetrahedra etc.

Here we focus our attention on Complex Quantum Network Manifolds (CQNMs) of dimension *d* constructed by gluing together simplices of dimension *d*. The CQNMs grow according to a non-equilibrium dynamics determined by the energies associated to its nodes, and have an emergent geometry, i.e. the geometry of the CQNM is not imposed a priori on the network manifold, but it is determined by its stochastic dynamics. Following a similar procedure as used in several other manuscripts^8^,^9^,^10^,^41^, one can show that the CQNMs characterize the time evolution of the quantum network states. In particular, each network evolution can be considered as a possible path over which the path integral characterizing the quantum network states can be calculated. Here we show that in *d *= 2 CQNMs are homogeneous and have an exponential degree distribution while the CQNMs are always scale-free for *d *> 2. Therefore for *d *= 2 the degree distribution of the CQNM has bounded fluctuations and is homogeneous while for *d *= 2 the CQNM has unbounded fluctuations in the degree distribution and its structure is dominated by hub nodes. Moreover, in CQNM quantum statistics emerges spontaneously from the network dynamics. In fact, here we define the generalized degrees of the 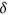-faces forming the manifold and we show that the average of the generalized degrees of the 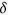-faces with energy 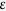 follows different statistics (Fermi-Dirac, Boltzmann or Bose-Einstein statistics) depending on the dimensionality 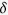 of the faces and on the dimensionality 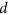 of the CQNM. For example in *d *= 2 the average of the generalized degree of the links follows a Fermi-Dirac distribution and the average of the generalized degrees of the nodes follows a Boltzmann distribution. In *d *= 3 the faces of the tetrahedra, the links and the nodes have an average of their generalized degree that follows respectively the Fermi-Dirac distribution, the Boltzmann distribution and the Bose-Einstein distribution.

Consider a 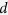-dimensional simplicial complex formed by gluing together simplices of dimension 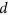, i.e. a triangle for *d *= 2, a tetrahedron for *d *= 3 etc. A necessary requirement for obtaining a discretization of a manifold is that each simplex of dimension 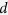 can be glued to another simplex only in such a way that the (*d*−1)-faces formed by (*d*−1)-dimensional simplices (links in *d *= 2, triangles in *d *= 3, etc.) belong at most to two simplices of dimension 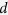.

Here we indicate with 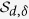 the set of all 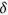-faces belonging to the 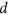-dimensional manifold with *δ *< *d*. If a (*d*−1)-face 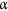 belongs to two simplices of dimension 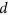 we will say that it is “saturated” and we indicate this by an associated variable *ξ*_*α*_ with value *ξ*_*α *_= 0; if it belongs to only one simplicial complex of dimension 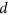 we will say that it is “unsaturated” and we will indicate this by setting *ξ*_*α *_= 1.

The CQNM is evolving according to a non-equilibrium dynamics described in the following.

To each node *i *= 1, 2…, *N* an *energy* of the node 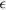
_*i*_ is assigned from a distribution 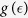. The energy of the node is quenched and does not change during the evolution of the network. To every 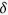-face 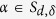 we associate an *energy* 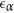 given by the sum of the energy of the nodes that belong to the face *α*,


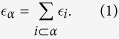


At time *t *= 1 the CQNM is formed by a single 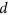-dimensional simplex. At each time *t *> 1 we add a simplex of dimension 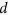 to an unsaturated (*d−*1)-face 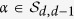 of dimension *d−*1. We choose this simplex with probability Π*α* given by


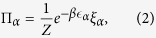


where *β* is a parameter of the model called *inverse temperature* and *Z* is a normalization sum given by


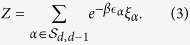


Having chosen the (*d−*1)-face *α*, we glue to it a new *d*-dimensional simplex containing all the nodes of the (*d−*1)-face *α* plus the new node *i*. It follows that the new node *i* is linked to each node *j* belonging to *α*.

In Fig. 1 we show few steps of the evolution of a CQNM for the case *d *= 2, while in Fig. 2 we show examples of CQNM in *d *= 2 and in *d *= 3 for different values of *β*.

From the definition of the non-equilibrium dynamics described above, it is immediate to show that the network structure constructed by this non-equilibrium dynamics is connected and is a discrete manifold.

Since at time *t* the number of nodes of the network manifold is *N *= *t *+ *d*, the evolution of the network manifold is fully determined by the sequence 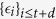, and the sequence 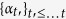, where 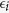, for *i *≤ *d *+ 1 indicates the energy of an initial node, while for 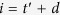 with 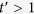 it indicates the energy of the node added at time 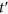, and where 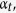 indicates the (*d*−1)-face to which the new 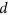-dimensional simplex is added at time 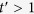.

The dynamics described above is inspired by biological evolutionary dynamics and is related to self-organized critical models. In fact the case 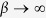 is dictated by an extremal dynamics that can be related to invasion percolation^28^,^45^, while the case *β *= 0 can be identified as an Eden model^46^ on a 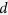-dimensional simplicial complex.

Here we call these network manifolds Complex Quantum Network Manifolds because using similar arguments already developed in^8^,^9^,^10^,^41^ it can be shown that they describe the evolution of Quantum Network States (see Methods and Supplementary Information for details). The quantum network state is an element of an Hilbert space *H*_*tot*_ associated to a simplicial complex of *N* nodes formed by gluing 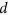-dimensional simplices (see Methods and Supplementary Information for details). The quantum network state 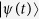 evolves through a Markovian non-equilibrium dynamics determined by the energies 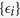 of the nodes. The quantity *Z*(*t*) enforcing the normalization of the quantum network state 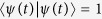 can be interpreted as a path integral over CQNM evolutions determined by the sequences 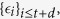 and 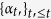. In fact we have


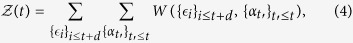


where the explicit expression of 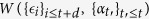 is given in the Supplementary Information. Moreover, *Z*(*t*) can be interpreted as the partition function of the statistical mechanics problem over the CQNM temporal evolutions. If we identify the sequences 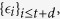 and 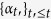, determining *Z*(*t*) with the sequences indicating the temporal evolution of the CQNM we have that the probability 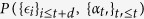 of a given CQNM evolution is given by


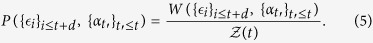


Therefore each classical evolution of the CQNM up to time *t* corresponds to one of the paths defining the evolution of the quantum network state up to time *t*.

A set of important structural properties of the CQNM are the generalized degrees 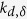 of its 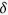-faces. Given a CQNM of dimension 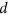, the generalized degree 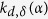 of a given 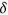-face 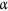, (i.e. 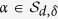) is defined as the number of 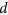-dimensional simplices incident to it. For example, in a CQNM of dimension *d *= 2, the generalized degree *k*_2,1_(*α*) is the number of triangles incident to a link 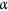 while the generalized degree *k*_2,0_(*α*) indicates the number of triangles incident to a node *α*. Similarly in a CQNM of dimension *d *= 3, the generalized degrees *k*_3,2,_ *k*_3,2_ and *k*_3,0_ indicate the number of tetrahedra incident respectively to a triangular face, a link or a node. If from a CQNM of dimension 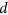 one extracts the underlying network, the degree *K*_*d*_(*i*) of node *i* is given by the generalized degree *K*_*d,*0_(*i*) of the same node 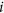 plus *d*−1, i.e.


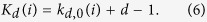


We indicate with 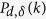 the distribution of generalized degrees *k*_*d,δ *_= *k*. It follows that the degree distribution of the network 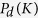 constructed from the *d*-dimensional CQNM is given by


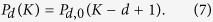


Let us consider the generalized degree distribution of CQNM in the case *β *= 0. In this case the new *d*-dimensional simplex can be added with equal probability to each unsaturated (*d*−1)-face of the CQNM. Here we show that as long as the dimension *d* is greater than two, i.e. *d *> 2, the CQNM is a scale-free network. In fact each 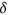-face, with 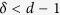, which has generalized degree 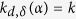, is incident to


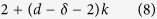


unsaturated (*d*−1)-faces. Therefore the probability 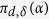 to attach a new 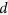-dimensional simplex to a 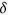-face 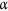 with generalized degree 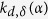 and with *δ *< d−1, is given by.


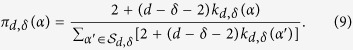


Therefore, as long as *δ *< d − 2, the generalized degree increases dynamically due to an effective “linear preferential attachment^24^”, according to which the generalized degree of a *δ*-face increases at each time by one, with a probability increasing linearly with the current value of its generalized degree. Since the preferential attachment is a well-known mechanism for generating scale-free distributions, it follows, by putting *δ *= 0, that we expect that as long as 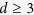 the CQNMs are scale-free. Instead, in the case *d *= 2, by putting *δ *= 0 it is immediate to see that the probability 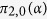 is independent of the generalized degree 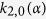 of the 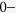
face (node) *α*, and therefore there is no “effective preferential attachment”. We expect therefore^17^ that the CQNM in *d *= 2 has an exponential degree distribution, i.e. in *d *= 2 we expect to observe homogeneous CQNM with bounded fluctuations in the degree distribution. These arguments can be made rigorous by solving the master equation^19^, and deriving the exact asymptotic generalized degree distributions for every *δ *< *d* (see Methods and Supplementary Information for details). For *δ *= *d *− 1 we find a bimodal distribution


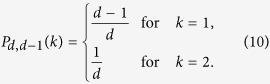


For 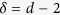 instead, we find an exponential distribution, i.e.


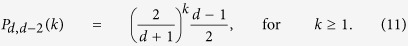


Therefore in *d *= 2, the CQNMs have an exponential degree distribution that can be derived from Eq. (11) and Eq. (7). Finally for 0 ≤ *δ *< *d *− 2 we have the distribution


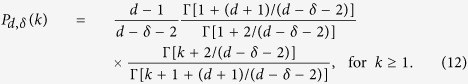


It follows that for 0 ≤ *δ *< *d* − 2 and 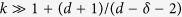 the generalized degree distribution follows a power-law with exponent 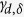, i.e.


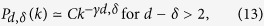


and


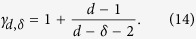


The distribution 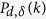 given by Eq. (12) is scale-free if an only if 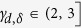. Using Eq. (14) we observe that for *d *≥ 3 and *δ *= 0 we observe that the distribution of generalized degrees 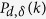 is always scale-free. Therefore the degree distribution 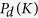 given by Eq. (7), for large values of the degree *K* and for *d *≥ 3 is scale-free and goes like


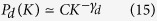


with


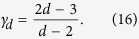


Therefore, for *d *= 3 the CQNMs have 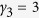 and for 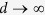 they have power-law exponent 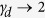.

These theoretical expectations perfectly fit the simulation results of the model as can been seen in Fig. 3 where the distribution of generalized degrees *P*_3,1_(*k*) and *P*_3,0_(*k*) observed in the simulations for *β *= 0 are compared with the theoretical expectations.

In the case *β *> 0 the distributions of the generalized degrees depend on the density 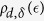 of 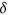-dimensional simplices with energy 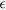 in a CQNM and are parametrized by self-consistent parameters called the chemical potentials, indicated as 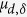 and defined in the Supplementary Information.

Here we suppose that these chemical potentials 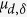 exist and that the density 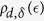 is given, and we find the self-consistent equations that they need to satisfy at the end of the derivation. Using the master equation approach^19^ we obtain that for 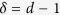 the generalized degree follows the distribution


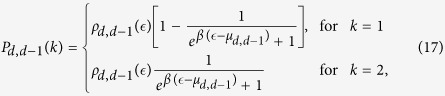


while for *δ *= *d *− 2 it follows


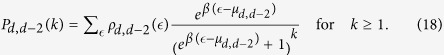


Finally for 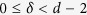 the generalized degree is given by


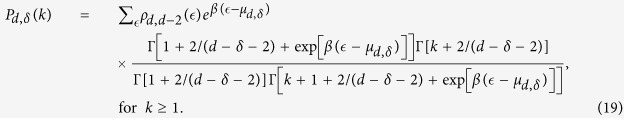


It follows that also for *β *> 0 the CQNMs in *d *> 2 are scale-free. Interestingly, we observe that the average of the generalized degrees of simplices with energy 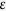 follows the Fermi-Dirac distribution for *δ *= *d *− 2, the Boltzmann distribution for *δ *= *d *− 2 and the Bose-Einstein distribution for *δ *= *d *> 2. In fact we have,


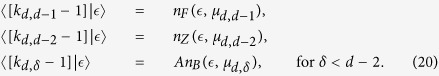


where 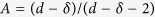, 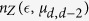 is proportional to the Boltzmann distribution and 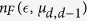, 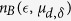 indicate respectively the Fermi-Dirac and Bose-Einstein occupation numbers^47^. In particular we have


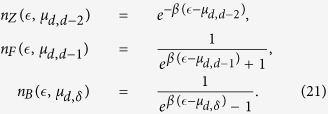


These results suggest that the dimension *d *= 3 of CQNM is the minimal one necessary for observing at the same time scale-free CQNMs and the simultaneous emergence of the Fermi-Dirac, Boltzmann and Bose-Einstein distributions. In particular in *d *= 3 the average generalized degree of triangles of energy 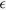 follows the Fermi-Dirac distribution, the average of the generalized degree of links of energy 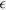 follows the Boltzmann distribution, while the generalized degree of nodes of energy 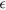 follows the Bose-Einstein distribution.

Finally the chemical potentials 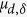, if they exist, can be found self-consistently by imposing the condition.


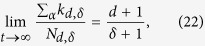


dictated by the geometry of the CQNM, which implies the following self-consistent relations for the chemical potentials 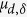



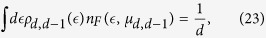



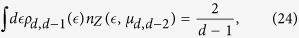



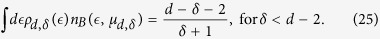


In Fig. 4 we compare the simulation results with the theoretical predictions given by Eqs. (20) finding very good agreement for sufficiently low values of the inverse temperature *β*. The disagreement occurring at large value of the inverse temperature *β* is due to the fact that the self-consistent Eqs. (23)–(25), 125, 126 do not always give a solution for the chemical potentials 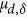. In particular the CQNM with 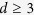 can undergo a Bose-Einstein condensation when Eq. (25) cannot be satisfied. When the transition occurs for the generalized degree with 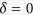, the maximal degree in the network increases linearly in time similarly to the scenario described in^27^.

In summary, we have shown that Complex Quantum Manifolds in dimension *d *> 2 are scale-free, i. e. they are characterized by large fluctuations of the degrees of the nodes. Moreover the 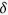-faces with 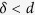 follow the Fermi-Dirac, Boltzmann or Bose-Einstein distributions depending on the dimensions 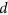 and 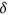. In particular for *d *= 3, we find that triangular faces follow the Fermi-Dirac distribution, links follow the Boltzmann distribution and nodes follow the Bose-Einstein distribution. Interestingly, we observe that the dimension *d *= 3 is not only the minimal dimension for having a scale-free CQNM, but it is also the minimal dimension for observing the simultaneous emergence of the Fermi-Dirac, Boltzmann or Bose-Einstein distributions in CQNMs.

## Methods

### Quantum network states

The Quantum Network State is an element of an Hilbert space *H*_*tot*_ associated to a simplicial complex formed by gluing 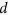-dimensional simplices of 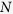 nodes. This Hilbert space is given by


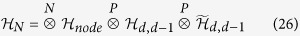


with 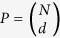 indicating the maximum number of (*d*−1)-dimensional simplices in a network of 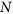 nodes. Here a Hilbert space 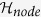 is associated to each possible node 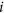 of the simplicial complex, and two Hilbert spaces 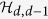 and 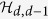 are associated to each possible (*d*−1)-dimensional simplex of a network of 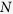 nodes. The Hilbert space 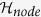 is the one of a fermionic oscillator of energy 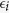, with basis 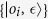, with 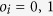. These states can be mapped respectively to the presence (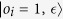) or the absence (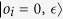) of a node 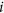 of energy 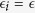 in the simplicial complex. We indicate with 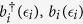 respectively the fermionic creation and annihilation operators acting in this space. The Hilbert space 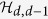 associated to a (*d*−1) simplex 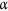 is the Hilbert space of a fermionic oscillator with basis 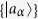, with 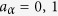. The quantum number 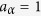 is mapped to the presence of the simplex 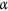 in the network while the quantum number 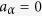 is mapped to the absence of such a simplex. We indicate with 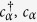 respectively the fermionic creation and annihilation operators acting in this space. Finally the Hilbert space 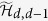 associated to a (*d*−1) simplex 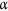 is the Hilbert space of a fermionic oscillator with basis 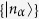, with 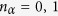. We indicate with 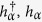 respectively the fermionic creation and annihilation operators acting in this space. The quantum number 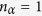 is mapped to a saturated 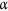 simplex, i. e. incident to two 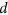-dimensional simplices, while the quantum number 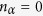 is mapped either to an unsaturated 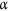 simplex (if also 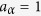) or to the absence of such a simplex (if 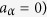.

A quantum network state can therefore be decomposed as


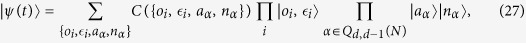


where with 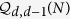 we indicated all the possible (*d*−1)-faces of the CQNM of 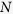 nodes.

We assume that the quantum network state follows a Markovian evolution as it has been proposed already in the literature^8^,^41^. In particular we assume that at time *t *= 1 the state is given by


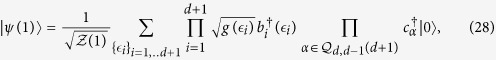


with 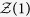 enforcing the normalization condition 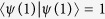. The quantum network state at each time 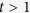 is updated according to the Markov chain


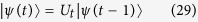


with the unitary operator 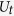 given by


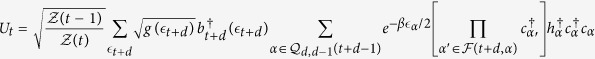


where 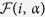 indicates the set of all the 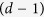-simplices 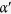 formed by the node 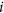 and a subset of the nodes in 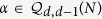. The quantity 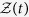 present in the definition of the unitary operator 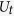 enforces the normalization condition 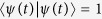 and can be interpreted as a path integral over CQNM evolutions determined by the sequence 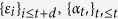 of the energy values 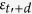 of the nodes added at time 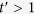 and the energy values 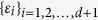, together with the sequence 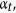 of the 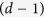-faces where the new 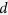-dimensional simplex is added at time 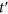. In fact we have.


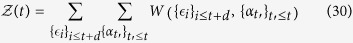


where the probability 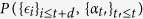 of a given CQNM evolution 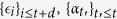 is given by


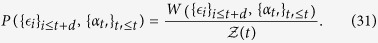


For the exact expression of 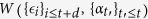 see the Supplementary Information.

### Generalized degree distribution for *β *= 0 

The average number of 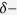
faces of a 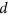-dimensional CQNM of generalized degree 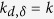 that are incident to the new 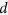-dimensional simplex at a given time *t* is given, for 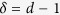, by


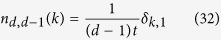


where 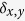 indicates the Kronecker delta while for *δ *< *d *− 1 is given by,


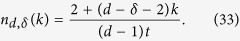


Using Eqs. (32), (33) and the master equation approach, it is possible to derive the exact distribution for the generalized degrees. We indicate with 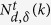 the average number of 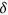-faces that at time 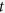 have generalized degree 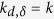. The master equation for 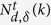 reads


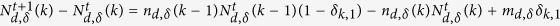


with *k *≥ 1. The master equation can be solved by observing that for large times 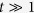 we have 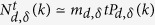 where 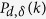 is the generalized degree distribution. In this way Eqs. (10)–(12), 85, 86 are obtained.

### Generalized degree distribution for *β *= 0

For *β *> 0 the probability 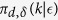 that a given 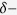
face of energy 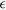 and generalized degree 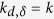 increases its generalized degree by one at time 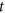 can be expressed in terms of self-consistent parameters 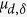 called *chemical potentials* and defined in the Supplementary Material. Using these probabilities the master equations can be written for the average number of 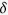-faces 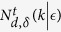 that at time 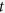 have generalized degree 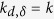 and energy 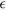. These equations can be solved similarly to the case *β *= 0 obtaining for the generalized degree distributions Eqs. (17)–(19), 108, 110.

## Additional Information

**How to cite this article**: Bianconi, G. and Rahmede, C. Complex Quantum Network Manifolds in Dimension *d*>2 are Scale-Free. *Sci. Rep.* **5**, 13979; doi: 10.1038/srep13979 (2015).

## Supplementary Material

Supplementary Information

## Figures and Tables

**Figure 1:**
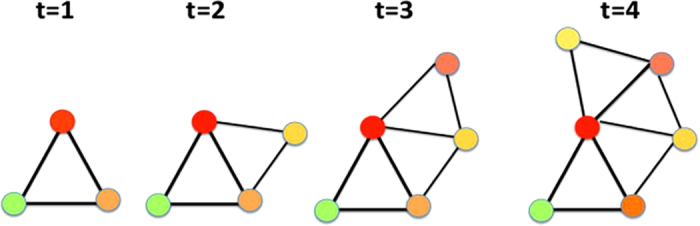
Evolution of the Complex Quantum Network Geometries in *d *= 2. Few steps of a possible evolution of the CQNM for *d *= 2. The nodes have different energies represented as different colours of the nodes. A link can be saturated (if two triangles are adjacent to it) or unsaturated (if only one triangle is incident to each). Starting from a single triangle at time *t *= 1, the CQNM evolves through the addition of new triangles to unsaturated links.

**Figure 2:**
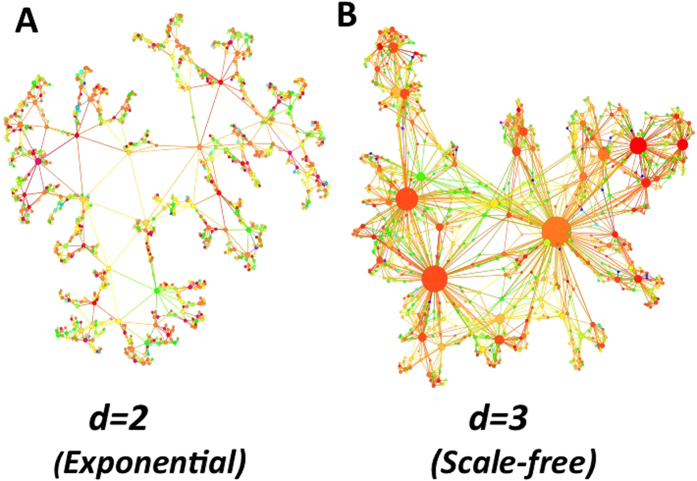
Visualization of Complex Quantum Network Geometries in dimensions *d *= 2,3. Visualization of CQNM with *d *= 2 (panel A) and *d *= 3 (panel B). The colours of the nodes indicate their energy 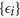 while their size indicates their degree 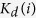. In *d *= 2 the degree distribution of the CQNMs is a convolution of exponentials, in *d *= 3 the CQNMs are scale-free and the presence of hubs is clearly observable from this visualization. The data shown are for CQNM with *N *= 10^3^ nodes, *β = *0.2 and Poisson distribution 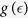 with average *z *= 5.

**Figure 3:**
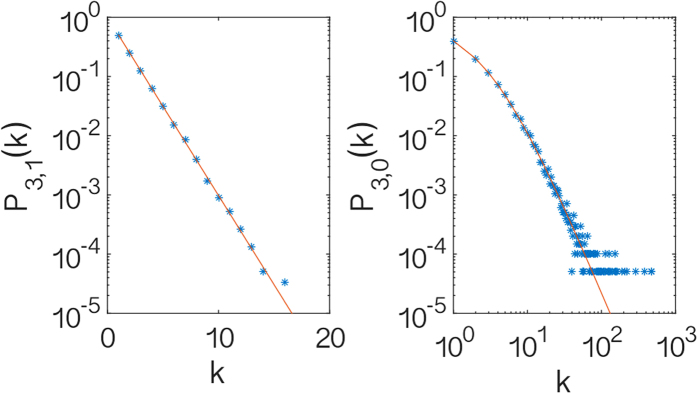
Distribution of the generalized degrees. The distribution of the (non-trivial) generalized degrees 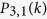 and 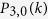 in dimension *d *= 3 are shown. The star symbols indicate the simulation results while the solid red line indicates the theoretical expectations given respectively by Eqs. (11) and (12). In particular we observe that 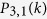 is exponential while 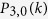 is scale-free implying that the CQNM in *d *= 3 is scale-free. The simulation results are shown for a single realization of the CQNM with a total number of nodes *N *= 2 × 10^4^.

**Figure 4:**
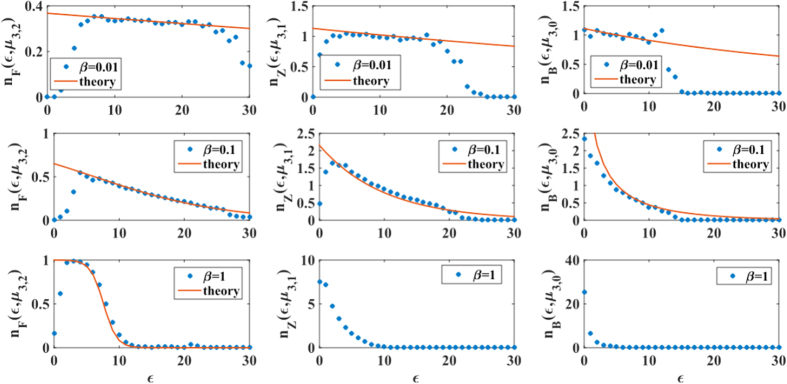
In *d *= 3 the average of the generalized degrees of faces, links, and nodes follow respectively the Fermi-Dirac, Boltzmann and Bose-Einstein distributions. The average of the generalized degrees of 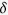-faces of energy 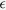, in a CQNM of dimension *d*=3, follow the Fermi-Dirac 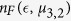, the Boltzmann 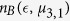 or the Bose-Einstein distribution 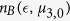 according to Eqs. (23)–(25), 125, 126 as long as the chemical potential 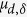 is well-defined, i.e. for sufficiently low value of the inverse temperature *β*. Here we compare simulation results over CQNM of *N *= 10^3^ nodes in *d *= 3 and theoretical results for *β *= 0.01, 0.1, 1. The CQNMs in the figure have a Poisson energy distribution 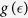 with average *z *= 5. The simulation results are averaged over 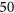 CQNM realizations.
