# Supplementary material for: Complex Quantum Network Manifolds in Dimension d > 2 are Scale-Free
Source: Sci Rep. 2015 Sep 10;5:13979. doi: 10.1038/srep13979 (PMC4564853; doi:10.1038/srep13979)
Supplement: Supplementary Information [file srep13979-s1.pdf]

Supplementary Material to "Complex Quantum Network Manifolds in Dimension $d > 2$ are Scale-Free"

Ginestra Bianconi[1] and Christoph Rahmede[2]

[1]School of Mathematical Sciences, Queen Mary University of London, London E1 4NS, United Kingdom

[2] Institute for Theoretical Physics, Karlsruhe Institute of Technology, 76128 Karlsruhe, Germany

I. INTRODUCTION

In this supplementary information we give the details of the derivation discussed in the main text. In Sec. II we define Complex Quantum Network Manifolds (CQNMs); in Sec. III we discuss the relation between the CQNM and the evolution of quantum network states; finally in Sec. IV we define the generalized degrees, and we derive the generalized degree distribution in the case $\beta = 0$ and $\beta > 0$.

II. COMPLEX QUANTUM NETWORK MANIFOLDS

Here we present the non-equilibrium dynamics of Complex Quantum Network Manifolds (CQNMs). This dynamics is inspired by biological evolution and self-organized models and generates discrete manifolds formed by simplicial complexes of dimension d. In particular CQNMs are formed by gluing d-simplices along $(d-1)$-faces, in order that each $(d-1)$-face belongs at most to two d-dimensional simplices.

Let us indicate with $\mathcal{S}_{d,\delta}$ the set of all δ-faces with $\delta < d$ belonging to the d-dimensional CQNMs. A $(d-1)$-face $\alpha \in \mathcal{S}_{d,d-1}$ is "saturated" if it belongs to two simplices of dimension d, whereas it is "unsaturated" if it belongs only to a single d-dimensional simplex. We will assign a variable $\xi_\alpha = 0, 1$ to each face $\alpha \in \mathcal{S}_{d,d-1}$, indicating either that the face is unsaturated ($\xi_\alpha = 1$) or that the face is saturated ($\xi_\alpha = 0$).

Moreover, to each node i we assign an *energy* ϵ_i drawn from a distribution $g(\epsilon)$ and quenched during the evolution of the network. To every δ-face $\alpha \in \mathcal{S}_{d,\delta}$ we associate an *energy* ϵ_α given by the sum of the energy of the nodes that belong to α,

$$\epsilon_\alpha = \sum_{i \subset \alpha} \epsilon_i \ . \tag{1}$$

At time $t = 1$ the CQNM is formed by a single d-dimensional simplex. At each time $t > 1$ we add a simplex of dimension d to an unsaturated $(d-1)$-face $\alpha \in \mathcal{S}_{d,d-1}$ chosen with probability Π_α given by

$$\Pi_\alpha = \frac{1}{Z} e^{-\beta \epsilon_\alpha} \xi_\alpha, \tag{2}$$

where β is a parameter of the model called *inverse temperature* and Z is a normalization

sum given by

$$Z = \sum_{\alpha \in \mathcal{S}_{d,d-1}} e^{-\beta\epsilon_\alpha} \xi_\alpha. \tag{3}$$

Having chosen the $(d-1)$-face α, we glue to it a new d-dimensional complex containing all the nodes of the face α plus the new node i. It follows that the new node i is linked to each node j belonging to α.

Since at time $t = 1$ the number of nodes in the CQNM is $N(1) = d+1$, and at each time we add a new additional node, the total number of nodes is $N(t) = t+d$. The CQNM evolution up to time t is fully determined by the sequences $\{\epsilon_i\}_{i \leq t+d}$, $\{\alpha_{t'}\}_{t' \leq t}$, where $\epsilon_{t'+d}$ indicates the energy of the node added to the CQNM at time $t' > 1$, ϵ_i with $i \leq d+1$ indicates the energy of an initial node i of the CQNM, and $\alpha_{t'}$ indicates the $(d-1)$-face to which the new d-dimensional complex is added at time t'. A similar dynamics for simplicial complexes of dimension $d = 2$ has been proposed in [1].

III. QUANTUM NETWORK STATES

A. The network Hilbert space

Following an approach similar to the one used in "Quantum Graphity" and related models [2–5], in this section we associate an Hilbert space $\mathcal{H}_{tot}$ to a simplicial complex of N nodes formed by gluing together d-dimensional simplices along $(d-1)$-faces. The Hilbert space $\mathcal{H}_{tot}$ is given by

$$\mathcal{H}_{tot} = \bigotimes^{N} \mathcal{H}_{node} \bigotimes^{P} \mathcal{H}_{d,d-1} \bigotimes^{P} \tilde{\mathcal{H}}_{d,d-1}, \tag{4}$$

with $P = \begin{pmatrix} N \\ d \end{pmatrix}$ indicating the maximum number of $(d-1)$-faces in a network of N nodes. Here an Hilbert space $\mathcal{H}_{node}$ is associated to each possible node i of the simplicial complex, and two Hilbert spaces $\mathcal{H}_{d,d-1}$ and $\tilde{\mathcal{H}}_{d,d-1}$ are associated to each possible $(d-1)-$face of a network of N nodes. The Hilbert space $\mathcal{H}_{node}$ is the one of a fermionic oscillator of energy ϵ, with basis $\{|o_i, \epsilon\rangle\}$, with $o_i = 0, 1$. We indicate with $b_i^\dagger(\epsilon), b_i(\epsilon)$ respectively the fermionic creation and annihilation operators acting on this space. The Hilbert space $\mathcal{H}_{d,d-1}$ associated to a $(d-1)$-face α is the Hilbert space of a fermionic oscillator with basis $\{|a_\alpha\rangle\}$,

with $a_\alpha = 0, 1$. We indicate with $c_\alpha^\dagger, c_\alpha$ respectively the fermionic creation and annihilation operators acting on this space. Finally the Hilbert space $\tilde{\mathcal{H}}_{d,d-1}$ associated to a $(d-1)$-face α is the Hilbert space of a fermionic oscillator with basis $\{|n_\alpha\rangle\}$, with $n_\alpha = 0, 1$. We indicate with $h_\alpha^\dagger, h_\alpha$ respectively the fermionic creation and annihilation operators acting on this space.

A quantum network state can therefore be decomposed as

$$|\psi(t)\rangle = \sum_{\{o_i,\epsilon_i,a_\alpha,n_\alpha\}} C(\{o_i,\epsilon_i,a_\alpha,n_\alpha\}) \prod_i |o_i,\epsilon_i\rangle \prod_{\alpha\in\mathcal{Q}_{d,d-1}(N)} |a_\alpha\rangle\,|n_\alpha\rangle\,, \tag{5}$$

where with $\mathcal{Q}_{d,d-1}(N)$ we indicate all the possible $(d-1)$-faces of a network of N nodes.

The node states $|o_i, \epsilon\rangle$ are mapped respectively to the presence ($|o_i = 1, \epsilon\rangle$) or the absence ($|o_i = 0, \epsilon\rangle$) of a node i of energy $\epsilon_i = \epsilon$ in the simplicial complex. The quantum state $|a_\alpha = 1\rangle$ is mapped to the presence of the $(d-1)-$face $\alpha \in \mathcal{S}_{d,d-1}$ in the network while the quantum state $|a_\alpha = 0\rangle$ is mapped to the absence of such a face. Moreover, when $a_\alpha = 1$, the quantum number $n_\alpha = 1$ is mapped to a saturated $(d-1)-$face α, i.e. α is incident to two d-dimensional simplices, while the quantum number $n_\alpha = 0$ is mapped either to an unsaturated $(d-1)-$face α (if also $a_\alpha = 1$) or to the absence of such a face (if $a_\alpha = 0$).

B. Markovian evolution of the quantum network states

As already proposed in the literature [2, 3], here we assume that the quantum network state follows a Markovian evolution. In particular we assume that at time $t = 1$ the state is given by

$$|\psi(1)\rangle = \frac{1}{\sqrt{\mathcal{Z}(1)}} \sum_{\{\epsilon_i\}_{i=1,..d+1}} \prod_{i=1}^{d+1} \sqrt{g(\epsilon_i)}\, b_i^\dagger(\epsilon_i) \prod_{\alpha\in\mathcal{Q}_{d,d-1}(d+1)} c_\alpha^\dagger\,|0\rangle\,, \tag{6}$$

where $\mathcal{Z}(1)$ is fixed by the normalization condition $\langle\psi(1)|\psi(1)\rangle = 1$. The quantum network state is updated at each time $t > 1$ according to the unitary transformation

$$|\psi(t)\rangle = U_t\,|\psi(t-1)\rangle \tag{7}$$

with the unitary operator U_t given by

$$U_t = \sqrt{\frac{\mathcal{Z}(t-1)}{\mathcal{Z}(t)}} \sum_{\epsilon_{t+d}} \sqrt{g(\epsilon_{t+d})}\, b_{t+d}^\dagger(\epsilon_{t+d}) \sum_{\alpha\in\mathcal{Q}_{d,d-1}(t+d-1)} e^{-\beta\epsilon_\alpha/2} \left[\prod_{\alpha'\in\mathcal{F}(t+d,\alpha)} c_{\alpha'}^\dagger\right] h_\alpha^\dagger c_\alpha^\dagger c_\alpha \tag{8}$$

where $\mathcal{F}(i,\alpha)$ indicates the set of all the $(d-1)$-faces α' formed by the node i and a subset of the nodes in $\alpha \in \mathcal{Q}_{d,d-1}(N)$ and $\mathcal{Z}(t)$ is fixed by the normalization condition

$$\langle \psi(t)|\psi(t)\rangle = 1. \tag{9}$$

C. Path integral characterizing the quantum network state evolution

The quantity $\mathcal{Z}(t)$ is a path integral over CQNM evolutions determined by the sequences $\{\epsilon_i\}_{i\leq t+d}, \{\alpha_{t'}\}_{t'\leq t}$. In fact, using the normalization condition in Eq. (9) and the evolution of the quantum network state given by Eqs. (7), (8) we get

$$\mathcal{Z} = \sum_{\{\epsilon_i\}_{i\leq t+d}} \sum_{\{\alpha_{t'}\}_{t'\leq t}} W(\{\epsilon_i\}_{i\leq t+d}, \{\alpha_{t'}\}_{t'\leq t}) \tag{10}$$

where $W(\{\epsilon_i\}_{i\leq t+d}, \{\alpha_{t'}\}_{t'\leq t})$ is given by

$$W(\{\epsilon_i\}_{i\leq t+d}, \{\alpha_{t'}\}_{t'\leq t}) \;=\; \prod_{i=1}^{t+d} g(\epsilon_i) \prod_{t'\leq t} a_{\alpha_{t'}}(t')(1-n_{\alpha(t')}(t')) e^{-\beta \sum_{\alpha\in\mathcal{Q}_{d,d-1}(t)} \epsilon_\alpha n_\alpha(t)}, \tag{11}$$

where the terms $a_\alpha(t)$ and $n_\alpha(t)$ that appear in Eq. (11) can be expressed in terms of the history $\{\alpha_{t'}\}_{t'\leq t}$ as

$$\begin{aligned} a_\alpha(t) \;&=\; \sum_{t'=2}^{t-1} \left(\sum_{\tilde{\alpha}\in\mathcal{F}(\alpha_{t'},t'+d)} \delta[\alpha,\tilde{\alpha}] \right) + \sum_{\tilde{\alpha}\in\mathcal{Q}_{d,d-1}(d+1)} \delta[\alpha,\tilde{\alpha}], \\ n_\alpha(t) \;&=\; \sum_{t'=2}^{t-1} \delta[\alpha_{t'},\alpha]. \end{aligned} \tag{12}$$

We note that $\mathcal{Z}(t)$ can also be interpreted as the partition function of the statistical mechanics problem over possible evolutions of CQNM. In fact, the CQNM evolution is determined by the sequences $\{\epsilon_i\}_{i\leq t+d}, \{\alpha_{t'}\}_{t'\leq t}$ where $\epsilon_{t'+d}$ indicates the energy of the node added at time $t' > 1$ and ϵ_i for $i \leq d+1$ indicates the energy of a node i in the CQNM at time $t=1$, and $\alpha_{t'}$ indicates the $(d-1)$-face to which we attach the new d-dimensional simplex at time t'. The probability $P(\{\epsilon_i\}_{i\leq t+d}, \{\alpha_{t'}\}_{t'\leq t})$ of a given evolution is given by

$$P(\{\epsilon_i\}_{i\leq t+d}, \{\alpha_{t'}\}_{t'\leq t}) = \frac{W(\{\epsilon_i\}_{i\leq t+d}, \{\alpha_{t'}\}_{t'\leq t})}{\mathcal{Z}(t)} \tag{13}$$

where $W(\{\epsilon_i\}_{i\leq t+d}, \{\alpha_{t'}\}_{t'\leq t})$ is given by Eq. (11) and $\mathcal{Z}(t)$ is fixed by the condition Eq. (9). This implies that the set of all classical evolutions of the CQNM fully determine the properties of the quantum network state evolving through the Markovian dynamics given by Eq. (7).

IV. GENERALIZED DEGREES

A. Definition of generalized degrees

A set of important structural properties of the CQNM are the generalized degrees $k_{d,\delta}(\alpha)$ of the δ-faces in a d-dimensional CQNM. Given a CQNM of dimension d, the generalized degree $k_{d,\delta}(\alpha)$ of a given δ-face α, (i.e. $\alpha \in \mathcal{S}_{d,\delta}$) is defined as the number of d-dimensional simplices incident to it. If we consider the adjacency tensor $\mathbf{a}$ of elements $a_\alpha = 1$ if the d-dimensional complex is part of the CQNM and otherwise zero, $a_\alpha = 0$, the generalized degree of a δ-face α' is given by

$$k_{d,\delta}(\alpha') = \sum_{\alpha|\alpha' \subset \alpha} a_\alpha. \tag{14}$$

For example, in a CQNM of dimension $d = 2$, the generalized degree $k_{2,1}(\alpha)$ is the number of triangles incident to a link α while the generalized degree $k_{2,0}(\alpha)$ indicates the number of triangles incident to a node α. Similarly in a CQNM of dimension $d = 3$, the generalized degrees $k_{3,2}$, $k_{3,1}$ and $k_{3,0}$ indicate the number of tetrahedra incident respectively to a triangular face, a link or a node.

B. Distribution of Generalized Degrees for $\beta = 0$

Let us define the probability $\pi_{d,\delta}(\alpha)$ that a new d-dimensional simplex is attached to a δ-face α. Since each d-dimensional simplex is attached to a random unsaturated $(d-1)-$face, and the number of such faces is $(d-1)t$, we have that for $\delta = d-1$

$$\pi_{d,d-1}(\alpha) = \begin{cases} \frac{1}{(d-1)t} & \text{for } k = 1, \\ 0 & \text{for } k = 2. \end{cases}$$

Let us now observe that each δ-face, with $\delta < d-1$, which has generalized degree $k_{d,\delta}(\alpha) = k$, is incident to

$$2 + (d - \delta - 2)k, \tag{15}$$

unsaturated $(d-1)$-faces.

In fact, is is easy to check that a $\delta-$face with generalized degree $k_{d,\delta} = k = 1$ is incident to $d-\delta$ unsaturated $(d-1)$-faces. Moreover, at each time we add to a δ-face a new d-dimensional

simplex, a number $d-\delta-1$ of unsaturated $(d-1)-$faces are added to the δ-face while a previously unsaturated $(d-1)$-face incident to it becomes saturated. Therefore the number of $(d-1)$-unsaturated faces incident to a δ-face of generalized degree $k_{d,\delta}=k$ follows Eq. (15). We have therefore that the probability $\pi_{d,\delta}(\alpha)$ to attach a new d-dimensional simplex to a δ-face α with $\delta<d-1$ and generalized degree $k_{d,\delta}(\alpha)$ is given by

$$\pi_{d,\delta}(\alpha)=\frac{2+(d-\delta-2)k_{d,\delta}(\alpha)}{\sum_{\alpha'\in\mathcal{S}_{d,\delta}}[2+(d-\delta-2)k_{d,\delta}(\alpha')]}, \tag{16}$$

where for large times $t\gg 1$,

$$\sum_{\alpha'\in\mathcal{S}_{d,\delta}}[2+(d-\delta-2)k_{d,\delta}(\alpha')]=2\begin{pmatrix} d \\ \delta \end{pmatrix}t+(d-\delta-2)\begin{pmatrix} d+1 \\ \delta+1 \end{pmatrix}t=(d-1)\begin{pmatrix} d \\ \delta+1 \end{pmatrix}t.$$

From Eq. (16) if follows that, as long as $\delta<d-2$, the generalized degree follows a "preferential attachment" mechanism [6–11].

Moreover, the average number $n_{d,\delta}(k)$ of δ-faces of generalized degree $k_{d,\delta}=k$ that increases their generalized degree by one at a generic time $t>1$, is given by

$$n_{d,\delta}(k)=m_{d,\delta+1}\pi_{d,\delta}(\alpha)|_{k_{d,\delta}(\alpha)=k} \tag{17}$$

where $m_{d,\delta+1}=\begin{pmatrix} d \\ \delta+1 \end{pmatrix}$ indicates the total number of $\delta-$faces incident to the d-dimensional simplex added at time t. This implies that for $\delta=d-1$, and large times $t\gg 1$, $n_{d,\delta}(k)$ is given by

$$n_{d,d-1}(k)=\frac{1}{(d-1)t}\delta_{k,1} \tag{18}$$

where $\delta_{x,y}$ indicates the Kronecker delta, while for $\delta<d-1$ it is given by

$$n_{d,\delta}(k)=\frac{2+(d-\delta-2)k}{(d-1)t}. \tag{19}$$

Using Eqs. (18) – (19) and the master equation approach [11], it is possible to derive the exact distribution for the generalized degrees. We indicate with $N^t_{d,\delta}(k)$ the average number of δ-faces that at time t have generalized degree $k_{d,\delta}=k$ during the temporal evolution of a d-dimensional CQNM. The master equation [11] for $N^t_{d,\delta}(k)$ reads

$$N^{t+1}_{d,\delta}(k)-N^t_{d,\delta}(k)=n_{d,\delta}(k-1)N^t_{d,\delta}(k-1)(1-\delta_{k,1})-n_{d,\delta}(k)N^t_{d,\delta}(k)+m_{d,\delta}\delta_{k,1} \tag{20}$$

with $k \geq 1$. Here $\delta_{x,y}$ indicates the Kronecker delta, and $m_{d,\delta} = \begin{pmatrix} d \\ \delta \end{pmatrix}$ is the number of δ-faces added at each time t to the CQNM. The master equation is solved by observing that for large times $t \gg 1$ we have $N^t_{d,\delta}(k) \simeq m_{d,\delta} t P_{d,\delta}(k)$ where $P_{d,\delta}(k)$ is the generalized degree distribution. For $\delta = d-1$ we obtain the bimodal distribution

$$P_{d,d-1}(k) = \begin{cases} \frac{d-1}{d}, & \text{for } k = 1 \\ \frac{1}{d} & \text{for } k = 2 \end{cases} .$$

For $\delta = d-2$ instead, we find an exponential distribution, i.e.

$$P_{d,d-2}(k) \ = \ \left(\tfrac{2}{d+1}\right)^k \tfrac{d-1}{2}, \ \text{for } k \geq 1 \ . \tag{21}$$

Finally for $0 \leq \delta < d-2$ we have the distribution

$$P_{d,\delta}(k) \ = \ \tfrac{d-1}{d-\delta-2} \tfrac{\Gamma[1+(d+1)/(d-\delta-2)]}{\Gamma[1+2/(d-\delta-2)]} \tfrac{\Gamma[k+2/(d-\delta-2)]}{\Gamma[k+1+(d+1)/(d-\delta-2)]}, \ \text{for } k \geq 1. \tag{22}$$

From Eq. (22) it follows that for $0 \leq \delta < d-2$ and $k \gg 1$ the generalized degree distribution follows a power-law with exponent $\gamma_{d,\delta}$, i.e.

$$P_{d,\delta}(k) \simeq Ck^{-\gamma_{d,\delta}} \ \text{for } d-\delta > 2, \tag{23}$$

and

$$\gamma_{d,\delta} = 1 + \frac{d-1}{d-\delta-2}. \tag{24}$$

Therefore the generalized degree distribution $P_{d,\delta}(k)$ given by Eq. (22) is scale-free, i.e. it has diverging second moment $\left\langle (k_{d,\delta})^2 \right\rangle$, as long as $\gamma_{d,\delta} \in (2,3]$. This implies that the generalized degree distribution is scale-free for

$$\delta < \frac{d-3}{2}. \tag{25}$$

C. Distribution of Generalized Degrees for $\beta > 0$

In the case $\beta > 0$ the distribution of the generalized degrees $P_{d,\delta}(k)$ are convolutions of the conditional probabilities $P_{d,\delta}(k|\epsilon)$ that δ-faces with energy ϵ have given generalized degree $k_{d,\delta} = k$. Here we derive the distribution of generalized degrees $P_{d,\delta}(k)$ for different values of δ and d as a function of the inverse temperature β. The procedure for finding these

distributions is similar for every value of δ.

First we will determine the master equations [11] for the average number $N^t_{d,\delta}(k|\epsilon)$ of δ-faces of energy ϵ that have generalized degree k at time t. Then we will solve these equations, imposing the scaling, valid in general for growing networks, given by $N_{d,\delta}(k|\epsilon) = m_{d,\delta}\rho_{d,\delta}(\epsilon)tP_{d,\delta}(k|\epsilon)$, where $\rho_{d,\delta}(\epsilon)$ is the probability that a δ-face has energy ϵ and $m_{d,\delta}$ are the number of δ-faces added at each time to the CQNM. The master equations will also depend on self-consistent parameters $\mu_{d,\delta}$ called *chemical potentials* that need to satisfy self-consistent equations for the derivation to hold.

Let us consider first the case $\delta = d-1$. The average number $N^t_{d,\delta}(k|\epsilon)$ of $\delta = (d-1)$-faces of energy ϵ that at time t have generalized degrees $k_{d,\delta} = k$ follows the master equation given by

$$\begin{aligned} N^{t+1}_{d,d-1}(k=2|\epsilon) - N^t_{d,d-1}(k=2|\epsilon) &= \frac{e^{-\beta\epsilon}}{Z}N^t_{d,d-1}(k=1|\epsilon), \\ N^{t+1}_{d,d-1}(k=1|\epsilon) - N^t_{d,d-1}(k=1|\epsilon) &= -\frac{e^{-\beta\epsilon}}{Z}N^t_{d,d-1}(k=1|\epsilon) + m_{d,d-1}\rho_{d,d-1}(\epsilon) \end{aligned} \tag{26}$$

where $\rho_{d,d-1}(\epsilon)$ is the probability that a $(d-1)$-face added to the network at a generic time $t \gg 1$ has energy ϵ and $m_{d,d-1} = \begin{pmatrix} d \\ d-1 \end{pmatrix} = d$ is the number of δ-faces added to the network at each time t. In order to solve this master equation we assume that the normalization constant $Z \propto t$ and we put

$$e^{-\beta\mu_{d,d-1}} = \lim_{t\to\infty}\frac{Z}{t}. \tag{27}$$

This is a self-consistent assumption that must be verified by the solution of Eqs. (26). Moreover we observe that at large times $N^t_{d,d-1}(k|\epsilon) \simeq m_{d,d-1}\rho_{d,d-1}(\epsilon)P_{d,d-1}(k|\epsilon)$. Here $P_{d,d-1}(k|\epsilon)$ indicates the asymptotic probability that a $(d-1)$-face α with energy ϵ has $k_{d,d-1}(\alpha) = k$. With these assumptions, we can solve Eqs. (26) finding

$$\begin{aligned} P_{d,d-1}(k=1|\epsilon) &= \rho_{d,d-1}(\epsilon)\frac{e^{\beta(\epsilon-\mu_{d,d-1})}}{e^{\beta(\epsilon-\mu_{d,d-1})}+1} = \rho_{d,d-1}(\epsilon)[1-n_F(\epsilon,\mu_{d,d-1})] \\ P_{d,d-1}(k=2|\epsilon) &= \rho_{d,d-1}(\epsilon)\frac{1}{e^{\beta(\epsilon-\mu_{d,d-1})}+1} = \rho_{d,d-1}(\epsilon)n_F(\epsilon,\mu_{d,d-1}), \end{aligned} \tag{28}$$

where $n_F(\epsilon,\mu_{d,d-1})$ is the Fermi-Dirac occupation number with chemical potential $\mu_{d,d-1}$, i.e.

$$n_F(\epsilon,\mu_{d,d-1}) = \frac{1}{e^{\beta(\epsilon-\mu_{d,d-1})}+1}. \tag{29}$$

Using Eqs. (28), and performing the average $\langle k_{d,d-1}|\epsilon\rangle$ of the generalized degree $k_{d,d-1}$ over $(d-1)$-faces of energy ϵ, one can easily find that

$$\langle [k_{d,d-1}-1]|\epsilon\rangle = \frac{1}{e^{\beta(\epsilon-\mu_{d,d-1})}+1} = n_F(\epsilon,\mu_{d,d-1}). \tag{30}$$

This result shows that the average of generalized degrees of $(d-1)$-faces of energy ϵ is determined by the Fermi-Dirac statistics with chemical potential $\mu_{d,d-1}$.

Let us now consider the case $\delta = d-2$. In this case we assume that asymptotically in time we can define the chemical potential $\mu_{d,d-2}$ as

$$e^{\beta\mu_{d,d-2}} = e^{\beta\mu_{d,d-1}} \lim_{t\to\infty} \left\langle \frac{\sum_{\alpha\in\mathcal{Q}_{d,d-2}(t)} \sum_{\alpha'\in\mathcal{Q}_{d,d-1}(t)|\alpha\subset\alpha'} e^{-\beta(\epsilon_{\alpha'}-\epsilon_\alpha)}\xi_{\alpha'}\delta(k_{d,d-2}(\alpha),k)}{\sum_{\alpha\in\mathcal{Q}_{d,d-2}(t)}\delta(k_{d,d-2}(\alpha),k)} \right\rangle_k . \tag{31}$$

In this assumption, the master equations [11] for the average number $N^t_{d,d-2}(k|\epsilon)$ of $(d-2)$-faces with energy ϵ and generalized degree $k\geq 1$, read

$$\begin{aligned} N^{t+1}_{d,d-2}(k|\epsilon) - N^t_{d,d-2}(k|\epsilon) &= \frac{e^{-\beta(\epsilon-\mu_{d,d-2})}}{t} N^t_{d,d-2}(k-1|\epsilon)[1-\delta_{k,1}] - \frac{e^{-\beta(\epsilon-\mu_{d,d-2})}}{t} N^t_{d,d-2}(k|\epsilon) \\ &\quad + m_{d,d-2}\rho_{d,d-2}(\epsilon)\delta_{k,1}, \end{aligned} \tag{32}$$

where $m_{d,d-2} = d(d-1)/2$ is the number of $(d-2)-$faces added at each time t to the CQNM, $\rho_{d,d-2}(\epsilon)$ is the probability that such faces have energy ϵ, and $\delta_{x,y}$ indicates the Kronecker delta. In the large network limit $t \gg 1$ we observe that $N^t_{d,d-2}(k|\epsilon) \simeq t m_{d,d-2}\rho_{d,d-2}(\epsilon)P_{d,d-2}(k|\epsilon)$ where $P_{d,d-2}(k|\epsilon)$ indicates the probability that a $(d-2)-$face of energy ϵ has generalized degree k. Solving Eq. (32) we get,

$$P_{d,d-2}(k|\epsilon) = \rho_{d,d-2}(\epsilon)\frac{e^{\beta(\epsilon-\mu_{d,d-2})}}{\left[e^{\beta(\epsilon-\mu_{d,d-2})}+1\right]^k}, \tag{33}$$

for $k\geq 1$. Therefore, summing over all the values of the energy of the nodes ϵ we get the full degree distribution $P(k)$

$$P_{d,d-2}(k) = \sum_\epsilon \rho_{d,d-2}(\epsilon)\frac{e^{\beta(\epsilon-\mu_{d,d-2})}}{\left[e^{\beta(\epsilon-\mu_{d,d-2})}+1\right]^k}, \tag{34}$$

for $k \geq 1$. Using Eqs. (34), and performing the average $\langle k_{d,d-2}|\epsilon\rangle$ of the generalized degree $k_{d,d-2}$ over $(d-2)$-faces of energy ϵ, one can easily find that

$$\langle [k_{d,d-2}-1]|\epsilon\rangle = e^{-\beta(\epsilon-\mu_{d,d-2})} = n_Z(\epsilon,\mu_{d,d-2}), \tag{35}$$

where we have indicated with $n_Z(\epsilon,\mu_{d,d-2})$ the Boltzmann distribution

$$n_Z(\epsilon,\mu_{d,d-2}) = e^{-\beta(\epsilon-\mu_{d,d-2})}. \tag{36}$$

This result shows that the average of generalized degrees of $(d-2)$-faces of energy ϵ is determined by the Boltzmann statistics with chemical potential $\mu_{d,d-2}$.

Let us finally consider the case $\delta < d-2$. In this case we assume that asymptotically in time we can define the chemical potential $\mu_{d,\delta}$ given by

$$e^{\beta\mu_{d,\delta}} = e^{\beta\mu_{d,d-1}} \lim_{t\to\infty} \left\langle \frac{\sum_{\alpha\in\mathcal{Q}_{d,\delta}(t)} \sum_{\alpha'\in\mathcal{Q}_{d,d-1}(t)|\alpha\subset\alpha'} e^{-\beta(\epsilon_{\alpha'}-\epsilon_\alpha)}\xi_{\alpha'}\delta(k_{d,\delta}(\alpha),k)}{\sum_{\alpha\in\mathcal{Q}_{d,\delta}(t)}[k+2/(d-2-\delta)]\,\delta(k_{d,\delta}(\alpha),k)} \right\rangle_k . \tag{37}$$

Assuming that the chemical potential $\mu_{d,\delta}$ exists, the master equations [11] for the average number $N^t_{d,\delta}(k|\epsilon)$ of δ-faces with energy ϵ and generalized degree $k\geq 1$ read

$$\begin{aligned} N^{t+1}_{d,\delta}(k|\epsilon) - N^t_{d,\delta}(k|\epsilon) \;=\; & \frac{e^{-\beta(\epsilon-\mu_{d,\delta})}[k-1+2/(d-\delta-2)]}{t} N^t_{d,\delta}(k-1|\epsilon)[1-\delta_{k,1}] \\ & -\frac{e^{-\beta(\epsilon-\mu_{d,\delta})}[k+2/(d-\delta-2)]}{t} N^t_{d,\delta}(k|\epsilon) \\ & + m_{d,\delta}\rho_{d,\delta}(\epsilon)\delta_{k,1}, \end{aligned} \tag{38}$$

where $m_{d,\delta} = \begin{pmatrix} d \\ \delta \end{pmatrix}$ is the number of $\delta-$faces added at each time t to the CQNM, $\rho_{d,\delta}(\epsilon)$ is the probability that such faces have energy ϵ, and $\delta_{x,y}$ indicates the Kronecker delta. In the large network limit $t\gg 1$ we observe that $N^t_{d,\delta}(k|\epsilon)\simeq t m_{d,\delta}\rho_{d,\delta}(\epsilon)P_{d,\delta}(k|\epsilon)$,where $P_{k,\delta}(k|\epsilon)$ is the probability that a $\delta-$face of energy ϵ has generalized degree k. Solving Eq. (38) we get,

$$P_{d,\delta}(k) \;=\; \sum_\epsilon \rho_{d,d-2}(\epsilon) e^{\beta(\epsilon-\mu_{d,\delta})} \frac{\Gamma\left[1+2/(d-\delta-2)+\exp\left[\beta\left(\epsilon-\mu_{d,\delta}\right)\right]\right]\Gamma[k+2/(d-\delta-2)]}{\Gamma[1+2/(d-\delta-2)]\Gamma\left[k+1+2/(d-\delta-2)+\exp\left[\beta\left(\epsilon-\mu_{d,\delta}\right)\right]\right]}, \text{ for } k\geq 1. \tag{39}$$

Using Eqs. (39), and performing the average $\langle k_{d,\delta}|\epsilon\rangle$ of the generalized degree $k_{d,\delta}$ over δ-faces of energy ϵ, one can easily find that

$$\langle [k_{d,\delta}-1]|\epsilon\rangle \;=A\frac{1}{e^{\beta(\epsilon-\mu_{d,\delta})}-1} = A n_B(\epsilon,\mu_{d,\delta}), \tag{40}$$

where $A=(d-\delta)/(d-\delta-2)$ and where $n_B(\epsilon,\mu_{d,\delta})$ indicates the Bose-Einstein occupation number with chemical potential $\mu_{d,\delta}$, i.e.

$$n_B(\epsilon,\mu_{d,\delta}) = \frac{1}{e^{\beta(\epsilon-\mu_{d,\delta})}-1}. \tag{41}$$

This result shows that the average of generalized degrees of δ-faces of energy ϵ is determined by the Bose-Einstein statistics with chemical potential $\mu_{d,\delta}$. Finally the chemical potentials $\mu_{d,\delta}$, if they exist, can be found self-consistently by imposing the condition

$$\lim_{t\to\infty}\frac{\sum_{\alpha}k_{d,\delta}}{N_{d,\delta}}=\frac{\begin{pmatrix}d+1\\ \delta+1\end{pmatrix}}{\begin{pmatrix}d\\ \delta\end{pmatrix}}=\frac{d+1}{\delta+1}, \tag{42}$$

dictated by the geometry of the CQNM. In fact at each time t we add to the network $m_{d,\delta}=\begin{pmatrix}d\\ \delta\end{pmatrix}$ new δ-faces and we increases the sum of the generalized degree $k_{d,\delta}$ by the amount $\begin{pmatrix}d+1\\ \delta+1\end{pmatrix}$. Imposing Eq. (42) implies the following normalization constraints for the chemical potentials $\mu_{d,\delta}$,

$$\begin{array}{l}\int d\epsilon\rho_{d,d-1}(\epsilon)n_F(\epsilon,\mu_{d,d-1})=\frac{1}{d},\\ \int d\epsilon\rho_{d,d-1}(\epsilon)n_Z(\epsilon,\mu_{d,d-2})=\frac{2}{d-1},\\ \int d\epsilon\rho_{d,\delta}(\epsilon)n_B(\epsilon,\mu_{d,\delta})=\frac{d-\delta-2}{\delta+1},\quad \text{for}\quad \delta<d-2.\end{array} \tag{43}$$

For small values of β, these equations have a solution that converges for $\beta\to 0$ to the $\beta=0$ solution discussed in the previous subsection. As the value of β increases it is possible that the chemical potentials $\mu_{d,\delta}$ become ill-defined and do not exist. In this case different phase transitions can occur. For the case $d=2$ these transitions have been discussed in detail in [2]. For $d>2$ we observe that the network might undergo a Bose-Einstein phase transition for values of the inverse temperature for which Eq. (43) cannot be solved in order to find the chemical potential $\mu_{d,\delta}$. The detailed discussion of the possible phase transitions in CQNM is beyond the scope of this work and will be the subject of a separate publication.

D. Mean-field treatment of the case $\beta>0$

It is interesting to characterize the evolution in time of the generalized degrees using the mean-field approach [11]. This approach reveals other aspects of the model that are responsible for the emergence of the statistics determining the distribution of the generalized degrees. Let us consider separately the mean-field equations determining the evolution of

the generalized degrees of $\delta-$faces with $\delta = d-1, d-2$ or with $\delta < d-2$.

The $(d-1)$-faces can have generalized degree $k_{d,d-1}$ that can take only two values $k_{d,d-1} = 1, 2$. The indicator ξ_α of a $(d-1)-$face α with generalized degree $k_{d,d-1}(\alpha) = k$ is given by

$$\xi_\alpha = 2 - k_{d,d-1}(\alpha). \tag{44}$$

In fact for $k = 2$ the face is saturated and $\xi_\alpha = 0$ while for $k = 1$ the face is unsaturated, therefore $\xi_\alpha = 1$. The mean-field approach consists in neglecting fluctuations, and identifying the variable ξ_α (evaluated at time t for a $\delta-$face α arrived in the CQNM at time t_α) with its average $\xi_\alpha = \hat{\xi}_\alpha(t, t_\alpha)$ over all the CQNM realizations. The mean-field equation for $\hat{\xi}_\alpha$ is given by

$$\frac{d\hat{\xi}_\alpha}{dt} = -\frac{e^{-\beta\epsilon_\alpha}\hat{\xi}_\alpha}{Z}, \tag{45}$$

with initial condition $\hat{\xi}_\alpha(t_\alpha, t_\alpha) = 1$ where t_α is the time at which the $(d-1)-$face is added to the CQNM. The dynamical Eq. (45) is derived from the dynamical rules of the CQNM evolution. In fact, at each time one $(d-1)-$face is chosen with probability Π_α given by Eq. (2). This face becomes unsaturated and glued to the new d-dimensional simplex. Therefore at each time $\hat{\xi}_\alpha$ indicating the average of ξ_α decreases in time by an amount given by Π_α. Assuming that for large time t we have $Z \simeq e^{-\beta\mu_{d,d-1}}t$, where the chemical potential $\mu_{d,d-1}$ is defined in Eq. (27), it follows that the solution of the mean-field Eq. (45) is given by

$$\hat{\xi}_\alpha(t, t_\alpha) = \left(\frac{t_\alpha}{t}\right)^{e^{-\beta(\epsilon_\alpha - \mu_{d,d-1})}}. \tag{46}$$

The average of $\hat{\xi}_\alpha$ over all $(d-1)-$faces α with energy ϵ, i.e. $\left\langle \hat{\xi}_\alpha | \epsilon \right\rangle$ is given by

$$\left\langle \hat{\xi}_\alpha | \epsilon \right\rangle = \langle [2 - k_{d,d-1}] | \epsilon \rangle = \frac{1}{t}\int_1^t dt_\alpha \left(\frac{t_\alpha}{t}\right)^{e^{-\beta(\epsilon - \mu_{d,d-1})}} = \frac{e^{\beta(\epsilon - \mu_{d,d-1})}}{1 + e^{\beta(\epsilon - \mu_{d,d-1})}} + o(t^{-1}). \tag{47}$$

Therefore we obtain also in the mean field approximation, that the average generalized degree of $(d-1)-$faces with energy ϵ, for $t \gg 1$ satisfies,

$$\langle [k_{d,d-1} - 1] | \epsilon \rangle = \frac{1}{1 + e^{\beta(\epsilon_\alpha - \mu_{d,d-1})}} = n_F(\epsilon - \mu_{d,d-1}), \tag{48}$$

where $n_F(\epsilon, \mu_{d,d-1})$ indicates the Fermi-Dirac occupation number with chemical potential $\mu_{d,d-1}$.

Let us consider now the generalized degree of $(d-2)$-faces using the mean-field approximation. Assuming that the chemical potential $\mu_{d,d-2}$ defined in Eq. (31) is well defined, it is possible to write down the mean field equation for the average $\hat{k}_{d,d-2}(\alpha)$ of the generalized degree of the $(d-2)$-face α over CQNM realizations. The solution of this equation will provide the evolution of the average of the generalized degree of a $(d-2)-$ face α with energy ϵ_α at time t, given that the face α is added to the CQNM at time t_α, i.e. the solution will specify the function $\hat{k}_{d,d-2}(\alpha) = \hat{k}_{d,d-2}(t, t_\alpha)$. The mean-field equation is given by

$$\frac{d\hat{k}_{d,d-2}(\alpha)}{dt} = \frac{e^{-\beta(\epsilon_\alpha - \mu_{d,d-2})}}{t}, \tag{49}$$

with solution $\hat{k}_{d,d-2}(\alpha) = \hat{k}_{d,d-2}(t, t_\alpha)$ and initial condition $\hat{k}_{d,d-2}(t_\alpha, t_\alpha) = 1$. It follows that $k_{d,d-2}(t, t_\alpha)$ evolves in time as

$$\hat{k}_{d,d-2}(t, t_\alpha) = e^{-\beta(\epsilon_\alpha - \mu_{d,d-2})} \ln\left(\frac{t}{t_\alpha}\right) + 1. \tag{50}$$

The average $\langle k_{d,d-2} - 1|\epsilon\rangle$ of the generalized degree minus one of $(d-2)-$faces with energy ϵ is given by

$$\langle [k_{d,d-2} - 1]|\epsilon\rangle = \left\langle [\hat{k}_{d,d-2} - 1]|\epsilon\right\rangle = \frac{1}{t}\int_1^t dt_\alpha e^{-\beta(\epsilon - \mu_{d,d-2})} \ln\left(\frac{t}{t_\alpha}\right) = e^{-\beta(\epsilon - \mu_{d,d-2})} + \mathcal{O}(\ln t/t).$$

Therefore we obtain also in the mean field approximation, that the average generalized degree of $(d-2)-$faces with energy ϵ, for $t \gg 1$ satisfies,

$$\langle [k_{d,d-2} - 1]|\epsilon\rangle = e^{\beta(\epsilon_\alpha - \mu_{d,d-2})} = n_Z(\epsilon, \mu_{d,d-2}), \tag{51}$$

where $n_Z(\epsilon, \mu_{d,d-2})$ is proportional to the Boltzman distribution at temperature $T = 1/\beta$. Finally the mean-field equation for the generalized degrees of δ-faces with $\delta < d-2$, is given by

$$\frac{d\hat{k}_{d,\delta}(\alpha)}{dt} = \frac{e^{-\beta(\epsilon_\alpha - \mu_{d,\delta})}[\hat{k}_{d,\delta}(\alpha) + 2/(d-\delta-2)]}{t}, \tag{52}$$

where one considers the solution $\hat{k}_{d,\delta}(\alpha) = \hat{k}_{d,\delta}(t, t_\alpha)$ with initial condition $\hat{k}_{d,\delta}(t_\alpha, t_\alpha) = 1$. Here $\hat{k}_{d,\delta}$ indicates the average over CQNM realizations of the average degree of the $\delta-$face α, and $\mu_{d,\delta}$ is the chemical potential defined in Eq. (37). The mean-field solution of Eq. (52) is given by

$$\hat{k}_{d,\delta}(t, t_\alpha) - \frac{2}{d-\delta-2} = A\left(\frac{t}{t_\alpha}\right)^{e^{-\beta(\epsilon_\alpha - \mu_{d,\delta})}}, \tag{53}$$

where $A = 1 + 2/(d-\delta-2) = (d-\delta)/(d-\delta-2)$. The average $\langle [k_{d,d-2}-1]|\epsilon\rangle$ of the generalized degree minus one of the $(d-2)-$faces with energy ϵ is given by

$$\langle [k_{d,\delta}-1]|\epsilon\rangle = \left\langle [\hat{k}_{d,\delta}-1]|\epsilon\right\rangle = A\frac{1}{t}\int_1^t dt_\alpha \left[\left(\frac{t}{t_\alpha}\right)^{e^{-\beta(\epsilon-\mu_{d,\delta})}}-1\right] = A\frac{1}{e^{\beta(\epsilon-\mu_{d,\delta})}-1} + \mathcal{O}(t^{-1+e^{\beta(\epsilon-\mu_{d,\delta})}}).$$

Therefore we obtain also in the mean field approximation, that the average generalized degree of $\delta-$faces with energy ϵ, and $\delta < d-2$, for $t \gg 1$ satisfies,

$$\langle [k_{d,\delta}-1]|\epsilon\rangle = A\frac{1}{e^{\beta(\epsilon-\mu_{d,\delta})}-1} = An_B(\epsilon,\mu_{d,\delta}), \tag{54}$$

where $n_B(\epsilon,\mu_{d,\delta})$ is proportional to the Bose-Einstein distribution at temperature $T = 1/\beta$.
As a final remark we note that the mean-field Eqs. (45), (49), (52) can be written by the single equation

$$\frac{dy}{dt} = [a_i + (1-|a_i|)]e^{-\beta(\epsilon-\mu)}\frac{y^{|a_i|}}{t} \tag{55}$$

with $a_i = -1, 0, 1$.
In fact Eq. (45) is equal to Eq. (55) where $a_i = -1$ and $y = \hat{\xi}_\alpha$. Eq. (49) is equal to Eq. (55) with $a_i = 0$ and $y = \hat{k}_{d,d-1}$. Finally Eq. (52) is equal to Eq. (49) with $a_i = 1$ and $y = \hat{k}_{d,\delta} + 2/(d-\delta-2)$.

---

[1] Wu, Z., Menichetti, G., Rahmede, C., & Bianconi, G. Emergent network geometry. *Scientific Reports*, **5**, 10073 (2015).

[2] Bianconi, G., Rahmede, C., Wu, Z. arXiv preprint. arXiv:1503.04739 (2015).

[3] Antonsen, F. Random graphs as a model for pregeometry. *International Journal of Theoretical Physics*, **33**, 1189-1205 (1994).

[4] Konopka, T., Markopoulou, F., & Severini, S. Quantum graphity: a model of emergent locality. *Phys. Rev. D* **77**, 104029 (2008).

[5] Hamma, A., Markopoulou, F., Lloyd, S., Caravelli, F., Severini, S., & Markström, K. Quantum Bose-Hubbard model with an evolving graph as a toy model for emergent spacetime. *Phys. Rev. D* **81**, 104032 (2010).

[6] Barabási, A. -L. & Albert, R. Emergence of scaling in random networks. *Science* **286**, 509-512 (1999).

[7] Albert, R., & Barabási, A. -L. Statistical mechanics of complex networks. *Rev. Mod. Phys.* **74**, 47-97 (2002).

[8] Newman, M. E. J. *Networks: An introduction.* (Oxford University Press, Oxford, 2010).

[9] Boccaletti, S., Latora, V., Moreno, Y., Chavez, M., & Hwang, D. H. Complex networks: Structure and dynamics. *Phys. Rep.* **424**, 175-308 (2006).

[10] Caldarelli, G. *Scale-free networks:complex webs in nature and technology.* (Cambridge University Press, Cambridge, 2007).

[11] Dorogovtsev, S. N., & Mendes, J. F. F. *Evolution of networks: From biological nets to the Internet and WWW.* (Oxford University Press, Oxford, 2003).
